# Recent advances in gut microbiota metabolite regulation of hepatic pregnane X receptor

**DOI:** 10.3389/fimmu.2025.1692684

**Published:** 2025-11-24

**Authors:** Tong Lin, Yang Chen, Linquan Liu, Tiesong Wu, Yan Qian, Baofen Jin

**Affiliations:** 1 Department of Pharmacy, Fuyong People's Hospital of Baoan District, Shenzhen, China; 2 Department of Emergency, Shenzhen Longhua District Central Hospital, Shenzhen, China; 3 Department of Chronic Disease Management, The First Hospital of Hunan University of Chinese Medicine, Changsha, China; 4 Department of Pharmacy, Shenzhen Longhua District Central Hospital, Shenzhen, China; 5 Public Relations Section, Shenzhen Longhua District Central Hospital, Shenzhen, China

**Keywords:** pregnane X receptor, ligand-binding domain, microbiota-PXR axis, inflammatory modulation, chemotherapy resistant

## Abstract

The pregnane X receptor (PXR), a key hepatic nuclear receptor, exhibits a highly plastic ligand-binding domain (LBD) that recognizes diverse endogenous and exogenous ligands, contributing to interindividual variations in xenobiotic metabolism and toxic responses. Emerging studies on the gut-liver axis reveal that microbiota metabolites regulate hepatic PXR through dual mechanisms: (1) Direct ligand-receptor interactions, where secondary bile acids (e.g., 3-keto LCA, DCA) and indole-3-propionic acid (IPA) bind PXR-LBD via hydrogen bonding to induce conformational changes, subsequently upregulating CYP3A4/ABCB1 expression while inhibiting NF-κB-mediated inflammation and modulating bile acid homeostasis through crosstalk with the farnesoid X receptor (FXR); and (2) Epigenetic reprogramming, wherein short-chain fatty acids (SCFAs) such as butyrate enhance PXR transcription by inhibiting histone deacetylase (HDAC) activity and promoting histone acetylation (e.g., at H3K9/K14 residues), thereby increasing promoter accessibility. This epigenetic mechanism contrasts with the direct ligand-binding pathway by acting indirectly through chromatin remodeling. Dysregulated PXR signaling underlies bile acid imbalance, mitochondrial dysfunction, and chemoresistance, driving clinical development of interventions including probiotic modulation of LCA/DCA balance, triptolide-mediated PXR activation, and structure-based PXR-targeted drug design. These findings highlight the microbiota-PXR axis as a critical determinant of drug response heterogeneity and a promising therapeutic target for metabolic liver disorders and refractory malignancies.

## 1 Introduction

The pregnane X receptor (PXR), a critical member of the nuclear receptor superfamily, exhibits distinct structural and functional characteristics that underpin its broad biological roles (1). Comprising a highly variable ligand-binding domain (LBD) and a conserved DNA-binding domain (DBD), the receptor recognizes diverse ligands such as bile acids and pharmaceuticals through its hydrophobic pocket, while forming heterodimers with the retinoid X receptor (RXR) to bind target gene promoter regions (2, 3). Its predominant expression in the liver and intestines aligns with its core physiological functions in regulating drug-metabolizing enzymes (*e.g.*, CYP3A4, P-gp) and metabolic networks of endogenous substances, including cholesterol and bile acids (4). Upon ligand activation, PXR recruits coactivators to initiate downstream gene transcription, playing a dual role in detoxification, glucose-lipid homeostasis, and inflammatory modulation (5).

Gut microbiota metabolites represent complex products of host-microbial co-metabolism, categorized into seven functional groups based on origin and activity (6–9). SCFAs, primarily derived from dietary fiber fermentation (60% acetate, 20% propionate, 20% butyrate), serve as energy sources for colon cells (butyrate accounting for 90%) and modulate insulin sensitivity and immune balance via GPCR activation (e.g., GPR43) (10). Neuroactive substances (e.g., GABA, 5-HT) and tryptophan metabolites (indole derivatives) form the gut-brain signaling network (11, 12), while secondary bile acids and trimethylamine N-oxide (TMAO) participate in cholesterol metabolism and cardiovascular risk regulation. Notably, these metabolites maintain intestinal barrier integrity (e.g., butyrate promoting mucin secretion) (13) but may induce inflammation when excessive (e.g., H_2_S disrupting epithelial junctions) (14), with their dynamic equilibrium directly linked to metabolic disease pathogenesis (15). Other metabolites include gases (H_2_, CH_4_, CO_2_) that sustain anaerobic environments and energy cycles, amino acid fermentation products (e.g., cadaverine, phenols, H_2_S), and microbial-synthesized vitamins (K, B-complex) involved in coagulation, energy metabolism, and DNA synthesis (16, 17).

Recent studies reveal that gut microbiota metabolites regulate PXR activity through direct binding or epigenetic modifications, forming a three-dimensional “microbe-metabolite-host receptor” interaction network (18, 19). For instance, secondary bile acids act as dual ligands for PXR and farnesoid X receptor (FXR), while SCFAs may influence PXR transcriptional efficiency via histone deacetylase (HDAC) inhibition (20, 21). This review systematically examines such cross-regulatory mechanisms, aiming to elucidate the potential impact of microbial metabolites on personalized medicine—including microbial explanations for drug metabolism variability and microbiota-based interventions for therapeutic optimization—thereby offering new perspectives for precision medicine in metabolic diseases and oncology.

## 2 Liver diseases associated with PXR dysregulation

PXR dysfunction in the liver is a critical factor in lipid metabolic disorders (22, 23), as the receptor maintains lipid homeostasis through three primary mechanisms: inhibiting lipid synthesis by downregulating key enzymes such as stearoyl-CoA desaturase (SCD1) (24) and acetyl-CoA carboxylase (ACC) (25), promoting fatty acid β-oxidation by enhancing peroxisome proliferator-activated receptor α (PPARα) and PPARγ coactivator 1α (PGC1α) pathways (26), and regulating bile acid metabolism by modulating rate-limiting enzymes like cholesterol 7α-hydroxylase (CYP7A1) (27, 28). Unlike the farnesoid X receptor (FXR), which primarily maintains bile acid balance and suppresses lipid synthesis (29, 30), PXR also regulates the transcription and expression of drug-metabolizing enzymes and transporters, including uridine diphosphate glucuronosyltransferases (UGTs), ATP-binding cassette transporter B1 (ABCB1/MDR-1), and cytochrome P450 3A4 (CYP3A4) (31).

PXR dysfunction triggers a cascade of pathological events: reduced CYP3A4 expression leads to secondary bile acid accumulation, disrupting lipid oxidation-synthesis balance; in obesity, PXR inactivation decreases very low-density lipoprotein (VLDL) secretion, causing free fatty acid spillover into muscle tissues and exacerbating peripheral insulin resistance; in cholestatic liver diseases (32), PXR fails to induce efflux transporters like multidrug resistance-associated protein 2 (MRP2) and breast cancer resistance protein (BCRP), resulting in bile acid retention and mitochondrial dysfunction (33, 34). The dysregulation of PXR-mediated lipid metabolism exacerbates mitochondrial dysfunction through multiple mechanisms. Specifically, activation by pregnenolone-16α-carbonitrile (PCN) significantly downregulates critical mitochondrial proteins including proline dehydrogenase (Prodh), cytochrome c, and Usmg (35, 36), thereby impairing protein folding quality control and degradation pathways. In hepatocytes, PXR dysfunction is closely linked to hepatobiliary diseases, such as primary biliary cholangitis, where toxic bile acids like lithocholic acid (LCA) inhibit PXR activity, leading to deficient MRP2 and BCRP expression and further bile acid retention (37). In drug-induced liver injury (DILI), gut microbiota-PXR protective mechanisms fail, reducing detoxification capacity and causing drug metabolite accumulation (38). Interventions, such as probiotic modulation of bile acid ratios or natural agonists like triptolide, aim to restore PXR function (39).

**Figure 1:**
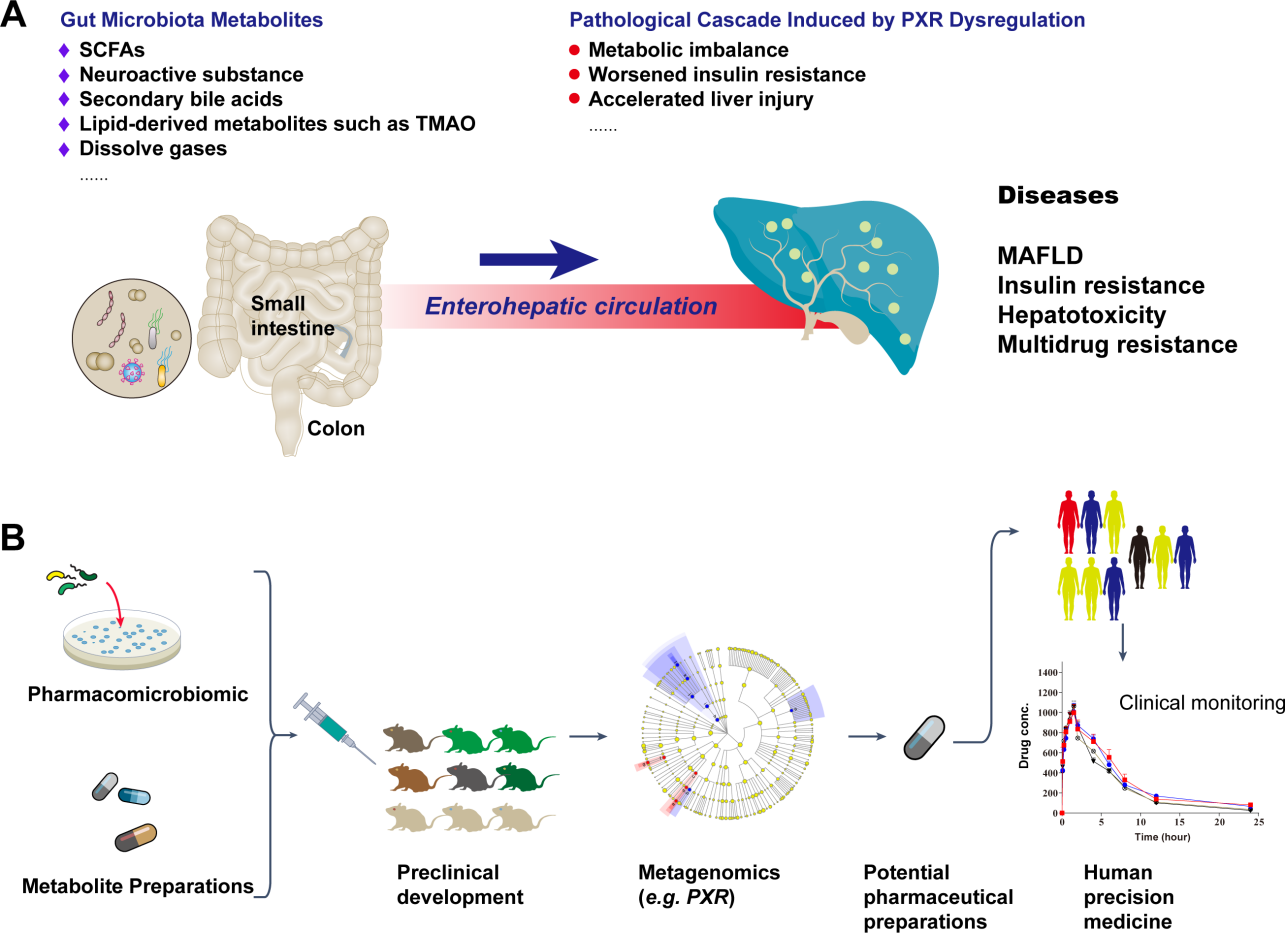
Gut microbiota metabolite-PXR axis in liver disease pathogenesis and the potential personalized therapeutic regimen development workflow. **(A)** Dysregulation of PXR: Impact of gut microbiota metabolites and gut-liver axis on liver metabolic disorders. **(B)** Potential roles of gut microbiota metabolites in hepatic metabolic transcription factors and a concise flowchart for personalized therapeutic development.

PXR dysfunction in the hepatobiliary system also influences chemotherapy resistance, as microbiota-derived metabolites alter drug-metabolizing enzyme profiles (40). For instance, reduced IPA diminishes PXR-mediated CYP3A4 induction, delaying irinotecan activation, while PXR overexpression may upregulate efflux transporters like MDR-1, creating multidrug resistance (41, 42). Studies show that tanshinone IIA can enhance sorafenib metabolism in hepatocellular carcinoma via PXR activation (43–45), highlighting the PXR-microbiota axis’s role in reversing chemoresistance (**Figure 1A**).

Gut microbiota and their metabolites have been clinically applied in gastrointestinal disease treatment (46, 47), with preclinical studies demonstrating their potential in improving hepatobiliary disorders (48, 49). Research has identified microbiota-derived metabolites that modulate PXR transcription through metagenomics, offering avenues for personalized therapy (50–52) (**Figure 1B**).

## 3 Recent advances in gut microbiota metabolite-mediated regulation of PXR activity

PXR, a ligand-activated nuclear receptor, primarily governs the inducible expression of xenobiotic-handling genes, encompassing biotransformation enzymes and drug transporters. Compared to other nuclear transcription factors, PXR assumes a pivotal role in modulating hepatic drug metabolism (53). In bile acid metabolism, PXR and FXR form a dynamically balanced regulatory loop: FXR regulates bile acid synthesis by inhibiting CYP7A1, while PXR promotes bile acid excretion by inducing CYP3A4. The two receptors achieve functional coordination through competitive binding to the promoter regions of common target genes (such as *ABCB11*), manifesting as bidirectional regulation of bile acid homeostasis in liver and biliary disease models (54). In drug metabolism, there exists substrate competition between PXR-induced CYP3A4 and FXR-regulated UGT1A1 (55, 56). For instance, rifampicin (a PXR agonist) can inhibit FXR-mediated bilirubin metabolism, leading to clinical drug interactions, which necessitates particular attention in the treatment of chronic liver disease. PXR and CAR exhibit significant synergistic effects in inducing phase II metabolic enzymes (such as UGT1A1, SULT2A1), but PXR demonstrates greater specificity in inducing CYP2B6 (57). The two receptors form a complex by sharing cofactors (such as RXRα) and initiate a coordinated detoxification response upon environmental toxin exposure. Notably, CAR is directly regulated by circadian clock genes, whereas PXR activation exhibits sustained induction properties (58). This time-dependent difference determines CAR’s dominant role in circadian metabolic fluctuations, while PXR is better suited for long-term drug exposure. As a signal integration hub, PXR forms a dynamic regulatory module by recruiting coactivators and corepressors, enabling simultaneous input from FXR, CAR, and PPARγ to achieve integrated regulation of metabolic pathways. In NAFLD models, the PXR-FXR-CAR tri-receptor network jointly determines the degree of lipid accumulation in hepatocytes by regulating the expression of lipid synthesis enzymes and transporters, with PXR activation partially reversing the lipid metabolic disorder caused by FXR deficiency (58, 59), highlighting its pivotal nodal role in disease networks.

**Figure 2:**
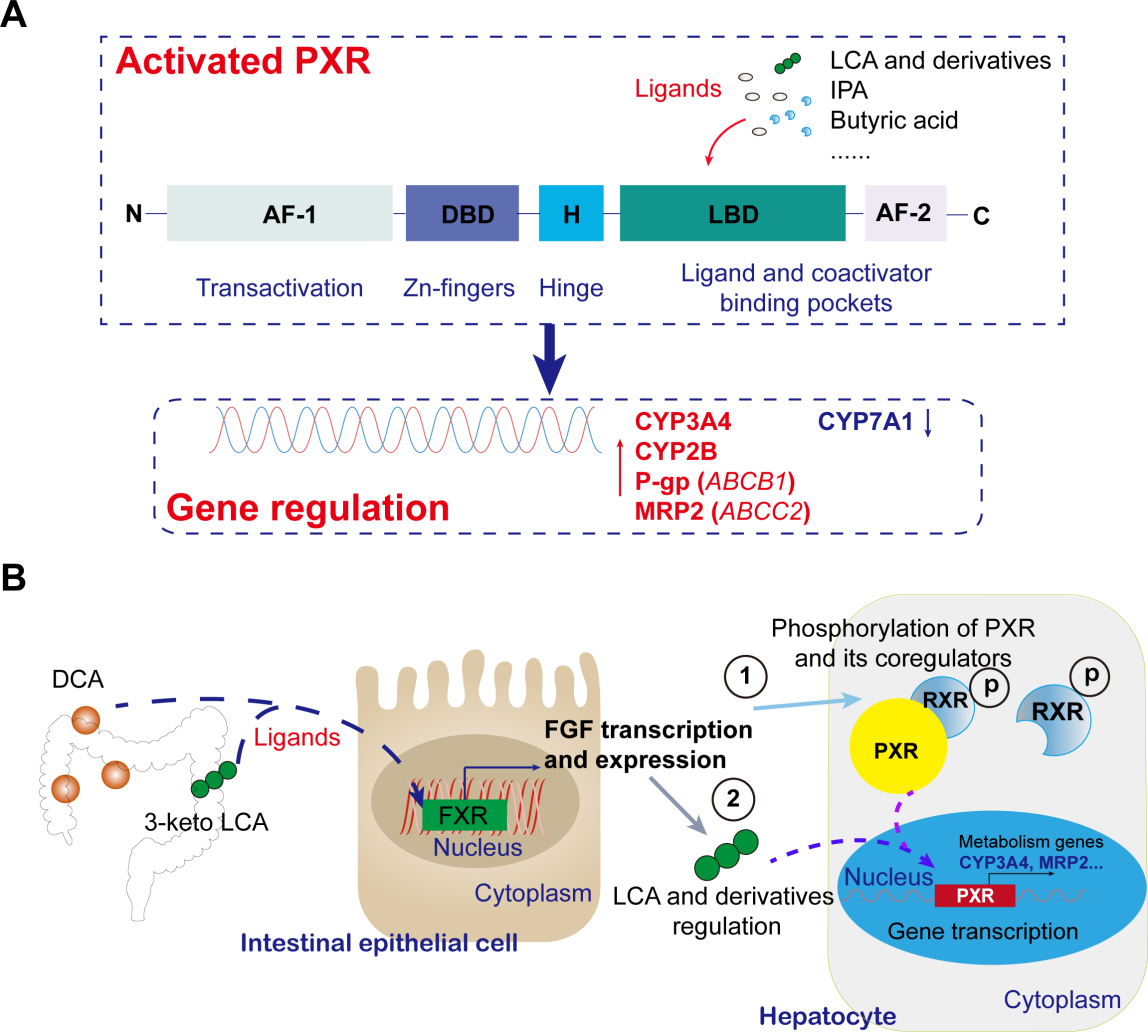
Recent mechanistic insights into gut microbiota metabolites modulating PXR transcriptional activity and functions. **(A)** Mechanism of activation of PXR by intestinally derived metabolites and its effects. In the direct activation pathway, microbial metabolites (e.g., 3-keto LCA, DCA, IPA) act as natural PXR ligands, binding via hydrogen bonds to conserved residues in the LBD. Activated PXR regulates target gene transcription, including upregulating *CYP3A4*, *ABCB1*, *ABCC2*, et al. expression and downregulating CYP7A1 expression. **(B)** Typical intestinally derived metabolites regulate the pathway of PXR in the liver. Microbial conversion of primary bile acids to secondary ones (e.g., DCA, 3-keto LCA) activates intestinal FXR, triggering FGF release. FGF modulating PXR through direct phosphorylation and indirect ligand regulation.

Recent studies have revealed that the long noncoding RNA HNF1A antisense 1 (HNF1A-AS1) exhibits dual regulatory functions in modulating CYP3A4 expression in Huh7 and HepG2 cells. Mechanistically, HNF1A-AS1 acts as an RNA scaffold to bind both protein arginine methyltransferase 1 and the pregnane X receptor (PXR), facilitating their interaction and thereby activating PXR and regulating CYP3A4 transcription through histone modifications. Consequently, small molecule-mediated epigenetic regulation holds promise as a novel biomarker for predicting individual differences in PXR-induced drug metabolism enzymes (60). Gut microbiota metabolites regulate PXR activity through two core pathways: direct ligand binding and epigenetic modulation (61). In the direct activation pathway, microbial-derived metabolites such as secondary bile acids (3-keto LCA, DCA) and indole-3-propionic acid (IPA) function as natural PXR ligands, establishing specific hydrogen bonding interactions with conserved residues including Arg410 and Gln285 within the LBD (50). Structural studies have revealed the remarkable adaptability of PXR’s binding pocket, exemplified by the 2.65 Å resolution crystal structure showing 17β-estradiol occupying only a localized region of the expansive cavity while bridging critical polar residues through its molecular framework (62). This unique binding mode underscores PXR’s exceptional capacity to accommodate diverse endobiotic ligands, a feature distinguishing it from other nuclear receptors (**Figure 2A**). Molecular dynamics simulations and *in vitro* assays have further demonstrated that carbamazepine (CBZ) likely acts as a PXR agonist, with Gln285 emerging as a pivotal interaction site (63). The structural plasticity of PXR-LBD enables heterodimerization with RXR upon activation, recruitment of coactivators like SRC-1, and binding to DR4 response elements in target gene promoters (e.g., *CYP3A4*, *ABCB1*) (64), thereby enhancing hepatic xenobiotic metabolism and suppressing intestinal NF-κB-mediated inflammation (**Figure 2A**). Notably, lithocholic acid derivatives mediate PXR-FXR crosstalk to regulate CYP7A1 activity (65), while indole compounds strengthen intestinal barrier function via the PXR-IL-10 axis (66, 67). Clinically, competition between metabolites (e.g., TMAO) and drugs for CYP3A4 binding may precipitate metabolic disturbances, highlighting the therapeutic implications of microbiota-PXR interactions.

While current research has firmly established that CYP3A4 enhancer methylation potently suppresses CYP3A4 expression via PXR-mediated mechanisms (with rifampicin-independent regulation) (68), the epigenetic modulation of PXR expression in hepatocytes by gut-derived SCFAs remains an emerging frontier (69). Mechanistic studies reveal that butyrate, a selective HDAC class I/II inhibitor (70), orchestrates PXR transcriptional activation may through two synergistic pathways: (1) site-specific acetylation of H3K9/K14 at the PXR promoter, and (2) GPR43-dependent sequestration of HDAC3 in the cytoplasm (71, 72). These findings are consistent with the broader paradigm that microbially derived SCFAs remodel hepatic chromatin architecture through HDAC inhibition (73), thereby simultaneously suppressing inflammatory cascades and potentiating PXR signaling (74). Nevertheless, critical knowledge gaps persist in deciphering the precise epigenetic orchestration between SCFA signaling and PXR regulatory networks in liver metabolism.

Microbial metabolite dysbiosis directly disrupts PXR function through three key pathways. First, LCA accumulation occurs due to gut microbiota imbalance, where LCA acts as a PXR antagonist (75), inhibiting lipid breakdown signaling and reducing the LCA/DCA ratio (normally PXR-activating), resulting in increase in SCD1 expression and exacerbating hepatic triglyceride deposition (76). Second, reduced IPA levels diminish PXR’s inhibitory effect on AKR1B10, thereby activating the ACC/SCD1 lipid synthesis pathway (77). Third, butyrate depletion impairs PXR function, as butyrate enhances PXR-RXRα dimerization by inhibiting HDAC3 (78). The carboxy-terminal domain of PXR contains a LBD that undergoes conformational changes upon binding specific ligands (e.g., rifampicin or aflatoxin), promoting PXR-RXRα heterodimer formation (79), cytoplasmic-nuclear translocation, and binding to direct repeat (DR) or estrogen receptor (ER) response elements in target gene promoters (80). Butyrate deficiency reduces PXR transcriptional activity, leading to impaired fatty acid oxidation (81). These mechanisms collectively demonstrate how microbiota-derived metabolites critically regulate PXR-dependent metabolic pathways.

Additionally, gut microbiota metabolites exert fine-tuned regulation of PXR activity through the FXR/FGF19 signaling axis, a key indirect regulatory network maintaining bile acid homeostasis (82). The molecular mechanism involves microbial conversion of primary bile acids into secondary bile acids (e.g., DCA, 3-keto LCA) that activate intestinal FXR, triggering FGF19/15 secretion (83). This hormone-like factor reaches the liver via portal circulation, binds to FGFR4-β-Klotho complexes, and suppresses CYP7A1 expression through RAS-RAF-MEK-ERK cascades (84). This process couples with PXR function through dual mechanisms: ERK-mediated phosphorylation of PXR/RXRα directly modulates transcriptional activity (85), while FGF19-maintained bile acid homeostasis indirectly regulates PXR-driven CYP3A4 expression by altering endogenous ligand concentrations (82, 86, 87) (**Figure 2B**). Under physiological conditions, this network forms a negative feedback loop where FXR-FGF19 inhibits excessive bile acid synthesis while PXR promotes detoxification (82). Pathologically, microbiota dysbiosis disrupts this balance, leading to bile acid accumulation and aberrant PXR activation that may cause drug metabolism disorders or hepatic inflammation (1, 88, 89). The elucidated “microbiota metabolite-FXR-FGF19-PXR” axis offers novel therapeutic targets (e.g., FGF19 biologics in clinical trials) and a framework for personalized medicine considering drug-microbiota interactions (**Figure 2B**). Notably, organ-specific PXR signaling exists: lithocholic acid activates hepatic PXR to upregulate CYP3A11 (90), while microbial-derived IPA preferentially stimulates intestinal PXR (Mdr1 upregulation) without hepatic effects, unlike hypericin which activates both tissues.

The dynamic interplay between TLR4/NF-κB and PXR pathways, mediated by gut microbiota-derived metabolites, constitutes a critical regulatory axis that maintains equilibrium between drug metabolism and inflammatory responses (91). Microbial immunomodulators such as peptidoglycan fragments (GlcNAc-MurNAc) activate NF-κB signaling through TLR2/NOD pathways (92), thereby inducing the production of key pro-inflammatory cytokines including IL-1β and TNF-α—a mechanism well-characterized in inflammatory bowel disease (IBD) and type 2 diabetes (93). This microbial-immune interaction is further modulated by direct molecular crosstalk: the NF-κB p65 subunit physically binds to PXR to inhibit its DNA-binding activity, while PXR activation in turn exerts negative transcriptional control over NF-κB (94). This bidirectional regulatory network exemplifies how microbiota metabolites fine-tune the delicate balance between xenobiotic processing and immune homeostasis.

Different microbiota metabolites exhibit differential regulatory effects on this network: SCFAs (e.g., butyrate) enhance histone acetylation at the PXR promoter region by inhibiting HDACs, partially counteracting NF-κB’s inhibitory effects, whereas trimethylamine N-oxide (TMAO) exacerbates metabolic disturbances by promoting hepatic sinus endothelial cell capillaryization and dysfunction, thereby modulating macrophage polarization (95). TMAO derived from gut microbiota exacerbates NAFLD progression by damaging the gut-liver axis, and targeting TMAO may offer alternative therapeutic strategies for NAFLD (95). Based on these findings, two intervention strategies have shown clinical potential: (1) using plant-derived bioactive compounds, such as baicalin, to selectively inhibit the TLR4/NF-κB pathway and restore PXR function (96); and (2) modulating microbial communities through fecal microbiota transplantation (FMT) or specific probiotics (e.g., butyrate-producing bacteria) to reestablish TLR4-PXR balance (97, 98) and mitigate the metabolic toxicity of xenobiotics like chemotherapeutic agents.

In conclusion, gut-derived metabolites directly or indirectly modulate PXR to regulate bile acid homeostasis and xenobiotic metabolism/transport, providing critical insights for personalized therapeutic strategies.

## 4 Gut-derived modulators of PXR activity

The gut microbiota orchestrates a complex regulatory network through secondary bile acids (e.g., lithocholic acid [LCA], deoxycholic acid [DCA]) and short-chain fatty acids (e.g., butyrate), which directly and indirectly modulate PXR activity. Secondary bile acids like LCA and DCA serve as endogenous PXR ligands, activating the receptor to upregulate drug-metabolizing enzymes (e.g., CYP3A4/2B) while suppressing CYP7A1 to maintain cholesterol homeostasis (99, 100). Notably, *Bacteroides stercoris* may influence drug pharmacokinetics by modulating GUDCA and GCDCA levels, which induce CYP3A1 expression in primary rat hepatocytes. Although known PXR-activating bile acids (including LCA, CDCA, DCA, and CA) showed no significant differences between groups in this study, existing research primarily focuses on unconjugated bile acids, with activation potency ordered as: 3-keto LCA > LCA > CDCA/DCA > CA (99, 100). Additionally, UDCA and TUDCA have been reported to activate PXR and induce CYP3A4 expression, though their precise mechanisms remain incompletely elucidated.

SCFAs, as microbial metabolites, enhance metabolic activity in liver organoids, including promoting CYP3A4 expression (101). Butyrate, in particular, contributes to PXR modulation by inhibiting HDACs (102), thereby enhancing PXR-mediated transcriptional regulation of glucose transport proteins GLUT2 (103), P-glycoprotein (ABCB1) (104), accelerating cholesterol metabolism and transport (104, 105). Post-gastrectomy studies reveal an adaptive LCA-PXR axis, where increased endogenous LCA levels and elevated *Bacteroides fragilis* abundance correlate with upregulated hepatic CYP3A11 expression (90, 106, 107), suggesting a compensatory protective mechanism against bile acid overload (108). Another notable modulator, indole-3-propionic acid (IPA), a tryptophan metabolite produced by *Clostridium* sp*orogenes* (109), acts as a PXR ligand to downregulate TNF-α and upregulate tight junction proteins, thereby maintaining gut barrier integrity (110), though its effects on CYP3A enzyme activity require further investigation.

The promiscuous nature of PXR, as a multi-ligand nuclear receptor, is underscored by its species-specific ligand-binding pocket—with only 75-80% amino acid sequence homology observed in the LBD across different species (111, 112). This structural divergence suggests significant interspecies variation in PXR ligand specificity (112, 113), a characteristic that further emphasizes the receptor’s pivotal role in bridging gut microbiota-derived signals (including bile acids, SCFAs, and other microbial metabolites) with host metabolic and detoxification pathways, as systematically documented in **Table 1**.

**Table 1:** PXR-modulating chemicals confirmed from microbial metabolites and their biological functions.

Category	Chemical	Regulatory role	Species	Key function	Reference
Direct regulatory	Butyrate	Activator	Human	Facilitating transcriptional activation and improving *ABCB1* mRNA stability	(104, 114, 115)
			Rat	Enhancing PXR - mediated transcriptional activation and alleviates liver cirrhosis	(102)
	LCA	Activator	Human	Hepatic PXR is activated by LCA to counteract the hepatotoxic effects of bile acid overload	(100)
				Activates intestinal PXR, which induces intestinal FGF19 expression to negatively feedback inhibit hepatic bile acid synthesis	(116)
			Mouse	Induces hepatic detoxification machinery and in a PXR-dependent manner	(117)
	3-keto LCA	Activator	Human	Alleviate the hepatotoxicity caused by bile acid overload and maintain cholesterol metabolic balance	(99, 100, 118, 119)
			Mouse	Alleviate the hepatotoxicity caused by bile acid overload and maintain cholesterol metabolic balance	(99, 100, 118, 119)
	DCA	Activator	Mouse	Upregulating drug-metabolizing enzymes such as CYP3A4/2B to facilitate hydroxylation detoxification of bile acids	(99)
	IPA	Activator	Human, mouse	Suppresses hepatic inflammatory cytokine production via PXR activation while enhancing intestinal barrier function to attenuate microbiota-derived toxin translocation to the liver	(110, 120)
	Skatole (3-methylindole)	Partial agonist and low affinity ligand	Human	Increases the expression and activity of CYP3A4 in human intestinal cells, but has no such effect in human hepatocytes	(119)
Indirect regulatoryPAMPs)	Lipopolysaccharides (LPS)	Repressor	Mouse	Induces NF-κB activation may suppress PXR expression, thereby compromising its regulatory capacity for drug-metabolizing enzymes (e.g., CYP3A4).	(121, 122)
	Peptidoglycan	Repressor	Mouse		(74, 92)

LCA, lithocholic acid; DCA, deoxycholic acid; IPA, Indole-3-propionic acid; PXR, pregnane X receptor; NF-κB, nuclear factor kappaB; CYP, Cytochrome P450. PAMPs, Pathogen-associated molecular patterns.

## 5 Regulation of PXR by clinically common drugs

PXR regulates numerous clinically used drugs beyond its prototype ligands (91, 123). Dexamethasone serves as a PXR activator in both mice and humans, as demonstrated by Pascussi et al. in 2001 and Yueh et al. in 2005 (124, 125). Notably, some drugs exhibit species-specific PXR activation patterns. For instance, phenobarbital enhances steroid receptor coactivator-1 binding to human PXR (hPXR) but fails to interact with mouse pregnane X receptor (mPXR) (126). The antifungal clotrimazole binds to hPXR at 10 mM concentrations, stimulating coactivator recruitment and enhancing PXR-target gene transcription (127), while showing weaker activation effects in rat and mouse PXR (128).

Recent studies (as of 2025) highlight PXR’s primary regulation over the following drug categories: CYP3A4 substrate drugs, where PXR preferentially induces CYP3A4 expression (accounting for approximately 30% of hepatic P450 enzymes) (129), accelerating the self-metabolic clearance of antibiotics like rifampicin and affecting the metabolic rates of warfarin and oral contraceptives. For example, PXR activation by rifampicin can increase warfarin metabolism, elevating the risk of anticoagulant therapy failure (130). CYP2B6 and CYP2C9 substrate drugs are regulated by PXR in synergy with constitutive androstane receptor (CAR), influencing the metabolism of antiepileptic drugs like phenobarbital, with clinical dosages adjusted based on receptor polymorphisms (e.g., *CAR* rs2307424) (131, 132). Transporter-dependent drugs are affected by PXR-induced expression of MDR1 (ABCB1) and MRP2 (ABCC2), regulating the enterohepatic circulation and biliary excretion of digoxin, as well as the hepatic concentration and myopathy risk of statins (via OATP1B1 transport). Bile acid-related drugs are indirectly regulated by PXR through modulation of bile acid metabolic enzymes (e.g., AKR1D1), influencing the generation of secondary bile acids and the efficacy of immunomodulatory drugs in liver cancer treatment (133). Glucocorticoids and anti-inflammatory drugs are affected by PXR polymorphisms (134), which can reduce glucocorticoid metabolic rates and impact the hepatoprotective effects of traditional Chinese medicine components like triptolide (135). PXR activation-induced drug interactions have become a clinical focus, such as the 47% increase in oral contraceptive failure rates when combined with St. John’s wort, prompting the FDA to require warning labels on related product inserts (136). Emerging research suggests that targeting PXR antagonists or modulating its signaling pathways may offer new strategies for personalized medicine.

## 6 Clinical significance and future perspectives

### 6.1 Microbial metabolite-mimetic drug development

IPA, a microbial indole metabolite, has been identified as a PXR activator, paving the way for novel drug development strategies. Through structural optimization, researchers have successfully designed the first non-cytotoxic PXR agonist, the lead compound FKK5/FKK6 (later named CVK003) (137). This compound directly binds to the PXR receptor, inducing PXR-target gene expression in cell cultures, human organoids, and mouse models. In humanized PXR transgenic mice, CVK003 significantly reduced pro-inflammatory cytokine levels (138). Further structural modification studies revealed that removing the benzenesulfonyl group shifted receptor binding specificity from PXR to the aryl hydrocarbon receptor (AhR), while losing PXR activation capability. Conversely, adding imidazopyridine maintained PXR binding and transcriptional activation (139, 140). These findings not only provide a theoretical basis for developing novel PXR modulators but also establish a research paradigm for understanding interactions between PXR and other xenobiotic-sensing transcription factors (141, 142).

### 6.2 Personalized therapy for liver diseases

PXR overactivation is associated with the progression of NAFLD, and microbiota-targeted regulation may offer a new therapeutic approach. Studies show that PXR dysfunction disrupts bile acid metabolic balance, exacerbating hepatic lipid accumulation and inflammatory responses, thereby promoting the transition from NAFLD to non-alcoholic steatohepatitis (NASH). Additionally, abnormal PXR activation can impair intestinal barrier function, promoting endotoxin translocation and further aggravating hepatic metabolic disorders. Modulating gut microbiota structure—such as increasing SCFA-producing probiotics—can restore bile acid metabolic homeostasis and indirectly inhibit excessive PXR activation, thereby reducing hepatic lipid peroxidation and insulin resistance. For example, prebiotics like inulin derivatives have been shown to reshape the gut microenvironment and enhance hepatic detoxification, offering new directions for personalized NAFLD treatment (143, 144). Future therapies combining PXR modulators with microbiota interventions may become key to overcoming NAFLD treatment bottlenecks (145). Probiotic interventions can also restore PXR function inhibition caused by antibiotics, improving metabolic variations of drugs like cyclosporine (146).

PXR’s dual role in liver disease progression and protection exhibits significant complexity. Regarding disease progression, PXR activation promotes hepatic lipid synthesis and fatty acid uptake while simultaneously inhibiting fatty acid β-oxidation, culminating in lipid accumulation and steatosis (147). Mechanistically, PXR drives this process through transcriptional upregulation of Solute carrier family 27 member 4 (SLC27A4), thereby accelerating NAFLD progression (148). In contrast, emerging evidence suggests protective roles for PXR modulation. Preclinical studies demonstrate that selective PXR modulators (e.g., hyodeoxycholic acid, HDCA) may improve metabolic function in early-stage disease (149). Furthermore, PXR activation mitigates drug-induced liver injury (DILI) through multifaceted mechanisms including enhanced detoxification regulation, anti-inflammatory effects, anti-apoptotic signaling, and improved bile acid excretion. Notably, PXR’s involvement in NAFLD remains controversial, with substantial discrepancies between preclinical and clinical findings. While PXR activation shows opposing effects on gluconeogenesis between rodents and humans (150), consistent evidence from HFHC diet-induced mouse models demonstrates that PXR activation triggers key NAFLD/NASH hallmarks including steatosis, inflammation, and lipotoxicity (22). This paradox may stem from PXR’s cell-specific expression pattern - as a hepatocyte-predominant nuclear transcription factor, its limited expression in Kupffer cells and hepatic stellate cells has resulted in insufficient research on its immune-modulatory roles in these cell populations. Collectively, these findings highlight the need for comprehensive studies to clarify PXR’s stage- and cell-specific functions in NAFLD/NASH pathogenesis. Future research should particularly address how PXR modulation in non-hepatocyte populations influences disease progression across different metabolic contexts.

The natural agonist ursolic acid activates PXR, significantly upregulating the phosphorylation of acetyl-CoA carboxylase (ACC), thereby inhibiting lipogenesis (151). This mechanism is closely linked to PXR’s transcriptional regulation of lipid metabolism genes, possibly involving indirect control of targets like stearoyl-CoA desaturase 1 (SCD1) (152). Additionally, excessive PXR activation is associated with NAFLD progression, and ursolic acid, as a PXR modulator, may offer new therapeutic strategies for metabolic liver diseases by balancing bile acid metabolism and improving intestinal barrier function. Future research could explore the synergistic effects of ursolic acid with other PPAR subtypes (e.g., PPARγ) to optimize its anti-lipidogenic efficacy (153). Mechanistic studies on the gut microbiota metabolite-PXR axis may further elucidate its role in metabolic liver disease progression.

Bile salt hydrolase (BSH) is a core enzyme in gut microbiota that converts primary bile acids to secondary ones, such as DCA, which indirectly affects PXR signaling by activating the FXR (154). Engineered BSH+ lactic acid bacteria may enhance bile acid metabolic efficiency, promoting PXR-dependent expression of hepatic detoxification enzymes (e.g., CYP3A4) and improving metabolic disorders (155). Recombinant BSH lactic acid bacteria not only improve gut colonization (with 2–3-fold upregulation of adhesion protein expression) but also regulate host immune microenvironments through SCFA secretion (156). Butyrate and other SCFAs have been shown to inhibit PXR transcriptional activity via HDAC inhibition.

### 6.3 Clinical prospects for PXR-targeted personalized therapy

In precision PXR modulation, building on the molecular design experience of the lead compound CVK003 (138), future strategies may include developing “smart-responsive” PXR modulators, such as pH/enzyme-sensitive prodrugs for targeted intestinal release (e.g., colon-specific delivery systems) and dual-functional molecules (e.g., PXR-FXR co-agonists) to synchronize bile acid synthesis and detoxification pathways (157). Ultrasound dynamics is a technology that utilizes ultrasonic energy to regulate drug delivery and enhance therapeutic efficacy. Its core mechanism lies in leveraging the physical effects of ultrasound—such as cavitation, mechanical vibration, and thermal effects—to alter tissue or cell membrane permeability, thereby facilitating targeted drug delivery or activating drug activity (158). When existing PXR modulators fail to achieve therapeutic effects, our department employs ultrasound dynamics to stimulate PXR modulators, thereby activating PXR function. This approach serves to modulate liver immunity, accelerate drug and bile acid metabolism, and ultimately alleviate liver immune diseases. Assessment models integrating fecal secondary bile acid profiles (DCA/LCA ratio), serum CYP3A4 activity, and gut microbiota BSH gene abundance could enable stratified treatment (159, 160). In host-microbiota co-intervention systems, optimizing BSH+ lactic acid bacteria colonization (via adhesion peptide integration), metabolic profiles (precisely regulating SCFA/DCA ratios), and immunomodulatory functions (e.g., carrying IL-10 anti-inflammatory genes) could provide personalized probiotic/prebiotic combinations (161, 162). Multi-omics technologies may predict PXR-responsive bacterial strains, analyze host-microbe interaction networks, and assess hepatocyte PXR pathway states, ultimately offering systematic solutions for PXR-targeted therapy.
